# Dynamic alignment changes during level walking in patients with dropped head syndrome: analyses using a three-dimensional motion analysis system

**DOI:** 10.1038/s41598-021-97329-w

**Published:** 2021-09-14

**Authors:** Tatsuya Igawa, Ken Ishii, Akifumi Suzuki, Hideto Ui, Ryunosuke Urata, Norihiro Isogai, Yutaka Sasao, Makoto Nishiyama, Haruki Funao

**Affiliations:** 1 Department of Orthopaedic Surgery, School of Medicine, International University of Health and Welfare, 852 Hatakeda, Narita City, Chiba 286-8520 Japan; 2 Department of Orthopaedic Surgery and Spine and Spinal Cord Center, International University of Health and Welfare Mita Hospital, 1-4-3, Mita, Minato-ku, Tokyo 108-8329 Japan; 3 Department of Rehabilitation, International University of Health and Welfare Mita Hospital, 1-4-3, Mita, Minato-ku, Tokyo 108-8329 Japan; 4 Department of Physical Therapy, School of Health Science, International University of Health and Welfare, 2600-1, Kitakanemaru, Ohtawara, Tochigi 323-8501 Japan

**Keywords:** Health care, Medical research

## Abstract

In patients with dropped head syndrome (DHS), cervical malalignment is one of the risk factors for impaired horizontal gaze and restrictions to ambulation. The characteristics of gait in patients with DHS have not been clarified biomechanically from the viewpoint of dynamic alignment and lower limb kinematics. This study aimed to clarify kinematic and kinetic differences during level walking in patients with DHS compared to the healthy elderly. Twelve patients with DHS and healthy elderly individuals performed level walking at a self-selected speed. Spatiotemporal, kinematic, and kinetic data were recorded using a three-dimensional motion analysis system. Statistical analysis was performed to compare these data between the two groups, respectively. Compared with the healthy elderly, stride length and peak hip-joint extension angle in patients with DHS were significantly shorter and smaller. The thorax was also significantly tilted backwards. Peak ankle-joint plantar-flexion moment was significantly smaller despite larger dorsiflexion angle compared with the healthy elderly. The walking of DHS patients demonstrated kinematic and kinetic characteristics of the lower limb joints and alignment of the thorax and pelvis corresponding to their short stride and walking speed.

## Introduction

Dropped head syndrome (DHS) is a relatively rare disease and a cervical kyphotic deformity with characteristic clinical features such as neck pain, restrictions to ambulation, and impaired horizontal gaze^1,2^. The gradual weakening of the paraspinal muscles ultimately results in the chin-on-chest deformity. In the relationship between the cervical and other parts of the spinal column, the pelvis and lower limbs change adaptively through a compensatory mechanism for global sagittal imbalance^3^. It has also been reported that an increase in neck flexion angle due to the use of a smartphone while walking affects neck load, walking speed, and muscle activity of the lower limbs^4^. Since the inclination of the head and trunk greatly affects the kinematics and kinematics of the lower limbs during walking^5^, DHS patients with abnormal cervical alignment might have characteristic gait features and different motor control compared to healthy individuals. We believe that it is important to include the lower limb joints in the dynamic assessment of DHS patients, as increased head flexion angle adversely affects postural control and could affect falls^6^. While the importance of assessing global sagittal balance in the practice of DHS patients has been suggested, the assessment of dynamic alignment remains imcomplete^7^. Recently, a new system has been developed to evaluate dynamic alignment using a three-dimensional (3D) motion analysis system, and there are a few case reports about gait in patients with DHS^8,9^. These papers describe the global sagittal imbalance by recording the coordinate points including C2, C7, T1 and S1 of DHS patients using a 3D motion analysis system. In the process of calculating their kinematics, the angle is calculated without taking into account complex movements such as lateral bending and rotation of the spinal column that appear during walking, so they are inaccurate values for trunk kinematics in patients with DHS. Understanding systemic postural control mechanisms might also be important in DHS patients who cannot control their head, because human posture is governed by what is known as top-down control, in which head stabilization control is performed prior to trunk stabilization^10^. Moreover, the therapeutic effect of DHS is higher in rehabilitation including whole body exercise than in training of cervical extensor muscle alone^11^. However, characteristics of the gait including the whole body such as the trunk and lower limbs in patients with DHS have not been clarified from the viewpoint of kinematics and kinetics. Elucidation of the systemic compensatory mechanism caused by dropped head may help to understand future rehabilitation programs. To our knowledge, this is the first report to confirm the gait characteristics of DHS patients calculated with consideration of 3D movement using a motion analysis system. The purpose of this study was to compare the gait kinematics and kinetics of DHS patients and healthy subjects, and to provide information that leads to the elucidation of gait control peculiar to DHS patients and information that is the basis of treatment.

## Methods

### Study design and participants

Approval was granted by the Ethics Committee of the International University of Health and Welfare Mita Hospital (No. 5-17-7). Written informed consent was obtained from all subjects before participation in this study. All procedures performed in studies involving human participants were in accordance with the ethical standards of the institutional and/or national research committee, and with the 1964 Helsinki declaration and its later amendments or comparable ethical standards. Additional written informed consent in the video for publication of this report was obtained from the patients. We prospectively assessed gait characteristics for twelve consecutive patients with DHS (mean age 73.5, range 66–79) at one hospital in Japan. Inclusion criteria were patients who have difficulty in lifting the head against center-of-gravity and horizontal gaze impairment and were diagnosed with idiopathic DHS. Exclusion criteria were patients with neuromuscular disease, inflammatory disease, mental illness, ankylosing spondylitis and history of surgery on spine or lower limb joints. Twelve age- and gender-matched healthy volunteers participated in this study for comparison (mean age 72.6, range 68–79).

### Data processing

**Figure 1:**
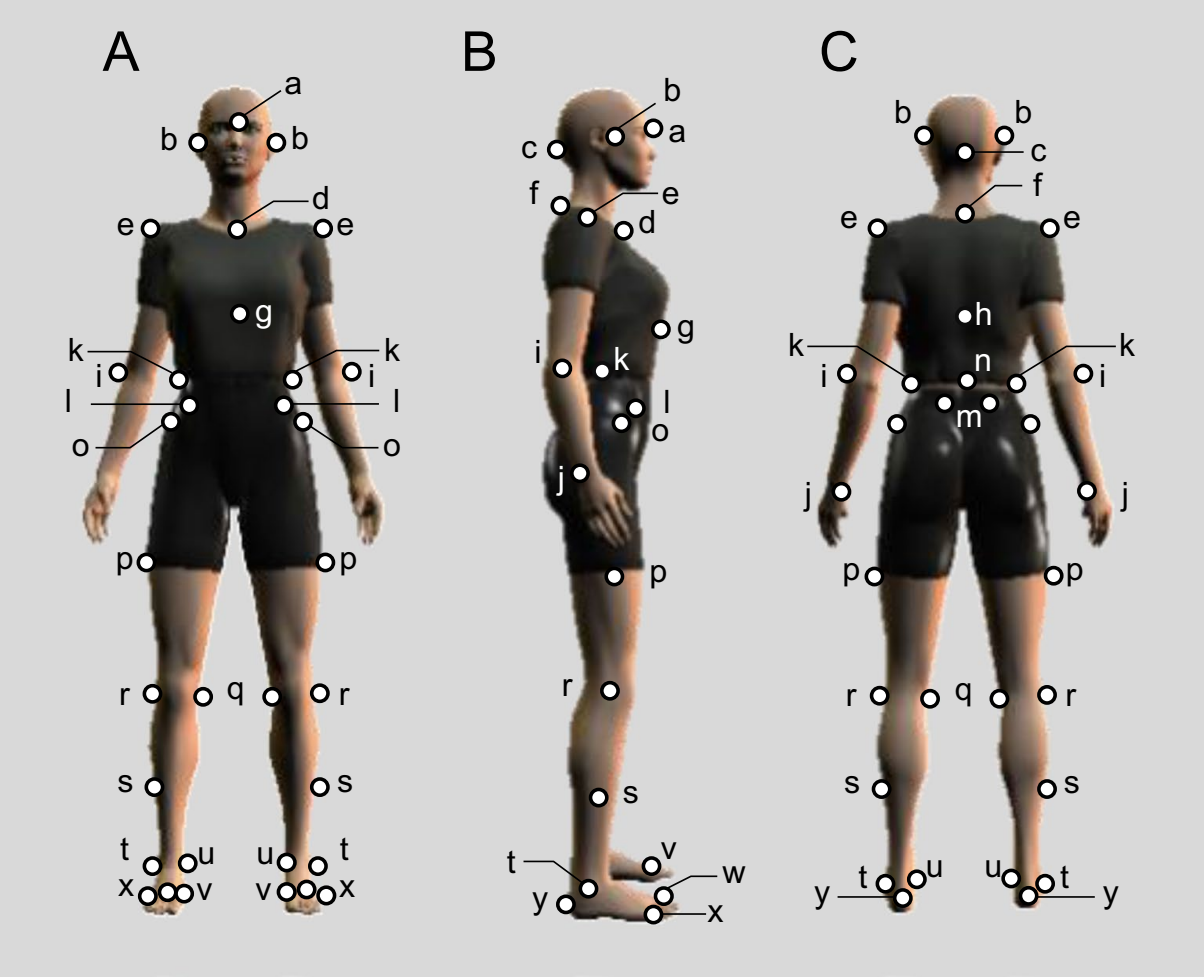
Forty-three reflective markers and trunk orthosis with joints providing resistive force. (**A**) Anterior view, (**B**) lateral view, and (**C**) posterior view. a, the bottom end of the center of the frontal bone; b, the articular tubercle; c, the external occipital protuberance; d, manubrium sterni; e, the acromion process; f, spinous process of the seventh cervical vertebra; g, xiphoid process; h, spinous process of the tenth thoracic vertebrae; i, the lateral epicondyle; j, ulnar styloid process; k, iliac crest; l, anterior superior iliac spine; m, posterior superior iliac spine; n, point between the fourth and fifth lumbar vertebrae; o, superior aspect of the greater trochanter; p, mid-point between the greater trochanter and lateral femoral condyles; q, medial knee; r, lateral knee; s, mid-point between the lateral knee joint line and lateral malleolus; t, lateral malleoli; u, medial malleoli; v, first metacarpophalangeal joints; w, second metacarpophalangeal joints; x, fifth metacarpophalangeal joints; y, heel.

A 3D motion analysis system was used to measure level walking. The system consisted of 10 MX cameras (Vicon Motion Systems Ltd, Oxford, UK) and six force plates (AMTI, Watertown, MA, USA). Three rows of two force plates were set up for data collection, and the kinematic and kinetic data were recorded at sample frequencies of 100 and 1000 Hz, respectively. To construct anatomical coordinate systems for each body segment, 43 reflective markers with a diameter of 14 mm were used as anatomical markers. These were landmarks consistent with our previous work and were attached according to the Helen Hayes and Plug-in-Gait marker protocols (Fig. 1)^12,13^. The subjects practiced walking for 2 min at a self-selected speed in the laboratory. Subjects were provided sufficient rest time before conducting the measurements. Gait measurement was performed in two gait cycles of the left and right lower limbs at a self-selected speed.

Joint kinematics and kinetics were analyzed using Visual 3D analytical software (C-motion, Germantown, MD, USA). The recorded data were low-pass filtered with respective cutoff frequencies of 6 and 18 Hz. Sagittal lower joint kinetics and kinematics in addition to movements of the thorax and pelvis were analyzed. The thorax segment was defined using the left and right iliac crest and shoulder markers. The seventh cervical vertebra, suprasternal notch, xiphoid process, and 10th thoracic vertebra markers were used as a cluster marker. The pelvic segment was defined using the CODA method from the anterior and posterior superior iliac spine markers. In the analysis, segments were modeled as rigid bodies, and internal joint moments were calculated using a link segment model where segments were connected at nodal points. The thoracic, pelvic, and hip angles were calculated based on absolute angles of the thoracic, pelvic, and thigh segments, respectively. The ankle and knee joint angles were calculated by the relative angle between the foot and shank segments and between the shank and thigh segments, respectively. Spatiotemporal parameters including walking speed, stride length, cycle time, and double support time (DST) and peak values for kinetic and kinematic parameters at the time of 1) loading response (LR), 2) single stance (SS), 3) pre-swing (PS), and 4) swing (SW) were extracted for analysis. The maximum distances between center of mass (COM) and center of pressure (COP) were calculated for the anterior–posterior (AP) and medio-lateral (ML) direction to evaluate the balance during walking, respectively. COM is based on weighted the average value of all segments; head, thoracic, humerus, radius, hand, pelvic, thigh, shank and foot. COP is based on the value of the ground reaction force (GRF). Since the relative positions of COM and COP in the anteroposterior direction are switched within one gait cycle, the distance when COM is farthest away from COP and the distance when COM is farthest behind COP were calculated, respectively. The ML distance is the average value of the maximum distances that appear once on each side during one gait cycle.

We defined these phases using the vertical component of the ground reaction force (GRF). Joint moments were normalized by body mass. In the analysis, the average value of the data of two gait cycles for each of the left and right lower limbs, that is, four gait cycles in total, was used as the representative value of the individual.

### Statistical analysis

Power analysis was performed using G*Power 3.1 (Heinrich Heine University, Düsseldorf, Germany). The sample size (12 patients) was determined to be able to detect a difference between both groups, assuming an effect size of 1.75, a type I error probability of 5%, and a type II error probability of 2% (i.e. power of 98%). Effect size was decided based on the result of the preliminary survey in which the walking speed of both groups was measured.

The demographic data and the other measurements were normally distributed according to the Kolmogorov–Smirnov test; therefore, a parametric statistical analysis was performed. Student's t-test was used for the data to compare the values of the two groups. A *p*-value of 0.05 was considered statistically significant. All statistical analyses were performed using SPSS for Windows version 25 software (IBM Corp., Armonk, NY, USA). Furthermore, statistical parametric mapping (SPM) unpaired t-tests were performed, comparing the mean lower limb kinematics and kinetics of each pattern to the respective mean lower limb kinematics and kinetics of the control group (α = 0.05). For each SPM t-test, SPM{t} was created by calculating the conventional univariate t- statistic at each point of the gait curve. These analyses were performed using open-source SPM1d code (vM.01.0003; www.spm1D.org) in Matlab.

## Results

The average age of the patients with DHS participating in this study was 73.5 years, and 83% of the patients were female. Radiographic sagittal alignment of the patients in a standing position showed in Table 1. C2–C7 sagittal vertical axis (SVA), which was measured as the distance between the perpendicular line from the center of the C2 vertebral body and posterosuperior corner of the C7 vertebra, was + 54.2 ± 16.0 mm. C7–S1 SVA, which was measured as the distance between the perpendicular line from the center of the C7 vertebral body and posterosuperior corner of sacrum, was − 11.4 ± 44.8 mm. C2–C7 angle was − 13.4 ± 26.4°, and lumbar lordosis was 42.4 ± 13.8°.

**Table 1:** Demographic data of the participants.

	DHS		Healthy		*p* value
	Mean	(SD)	Mean	SD	
Age (years)	73.5	(4.1)	72.6	(3.8)	0.574
Gender (F/M)	10/2		10/2		1.000
Height (cm)	151.4	(7.9)	156.5	(9.9)	0.180
Weight (kg)	46.9	(5.6)	54.3	(10.5)	**0.042**
Disease duration (months)	39.5	(42.5)			
NDI (/50)	13.6	(6.7)			
VAS of neck pain (mm)	53.8	(36.4)			
C2–C7 SVA (mm)	54.2	(16.0)			
C7–S1 SVA (mm)	− 11.4	(44.8)			
C2–C7 angle (deg)	− 13.4	(26.4)			
T1 slope (deg)	33.8	(18.1)			
Thoracic kyphosis (deg)	38.3	(16.6)			
Lumbar lordosis (deg)	42.4	(13.8)			
Pelvic incidence (deg)	50.9	(9.0)			
Pelvic tilt (deg)	29.1	(10.3)			

Bold figures indicate statistically significant *p* < 0.05.

*DHS* Dropped head syndrome, *NDI* neck disability index, *VAS* visual analogue scale, *SVA* sagittal vertical axis, *deg* degrees.

Spatiotemporal, kinematic, and kinetic parameters during level walking showed significant differences between the two groups. The walking speed of DHS patients was significantly slower than that of healthy subjects (*p* = 0.002), and stride length was also significantly shorter (*p* = 0.002) (Table 2). DST was significantly greater in patients with DHS (*p* = 0.026). The thorax angle in patients with DHS was significantly tilted backwards compared to healthy subjects (*p* = 0.011–0.021). Pelvis tended to be retroversion in patients with DHS, but no statistically significant difference in the pelvis angle was found between the two groups (Table 3).

**Table 2:** Comparison of spatiotemporal parameters between the two groups.

	DHS		Healthy		*p* value
	Mean	(SD)	Mean	(SD)	
Speed (m/s)	0.82	(0.15)	1.13	(0.20)	**0.002**
Stride length (m)	0.91	(0.13)	1.16	(0.19)	**0.002**
Cycle time (s)	1.12	(0.13)	1.04	(0.09)	0.118
Double support time (s)	0.26	(0.05)	0.22	(0.04)	**0.026**

Bold figures indicate statistically significant *p* < 0.05.

*DHS* Dropped head syndrome.

**Table 3:** Comparison of thorax and pelvis angles during walking between the two groups.

	DHS		Healthy		*p* value
	Mean	(SD)	Mean	(SD)	
**Thorax tilt angle (backward +)**					
Peak posterior tilt angle during LR	9.9	(4.0)	5.5	(3.4)	**0.008**
Peak posterior tilt angle during SS	10.0	(3.9)	5.8	(3.6)	**0.012**
Peak posterior tilt angle during PS	10.2	(4.2)	5.7	(3.7)	**0.012**
Peak posterior tilt angle during SW	10.3	(4.2)	6.1	(3.7)	**0.017**
**Pelvis tilt angle (backward +)**					
Peak posterior tilt angle during LR	− 1.6	(6.5)	− 6.3	(5.2)	0.066
Peak posterior tilt angle during SS	− 1.4	(6.4)	− 5.8	(5.0)	0.078
Peak posterior tilt angle during PS	− 1.5	(6.3)	− 5.9	(5.3)	0.082
Peak posterior tilt angle during SW	− 1.3	(6.4)	− 5.4	(5.4)	0.100

Bold figures indicate statistically significant *p* < 0.05. Unit is degree.

*DHS* Dropped head syndrome, *LR* loading response, *SS* single stance, *PS* pre-swing, *SW* swing.

**Figure 2:**
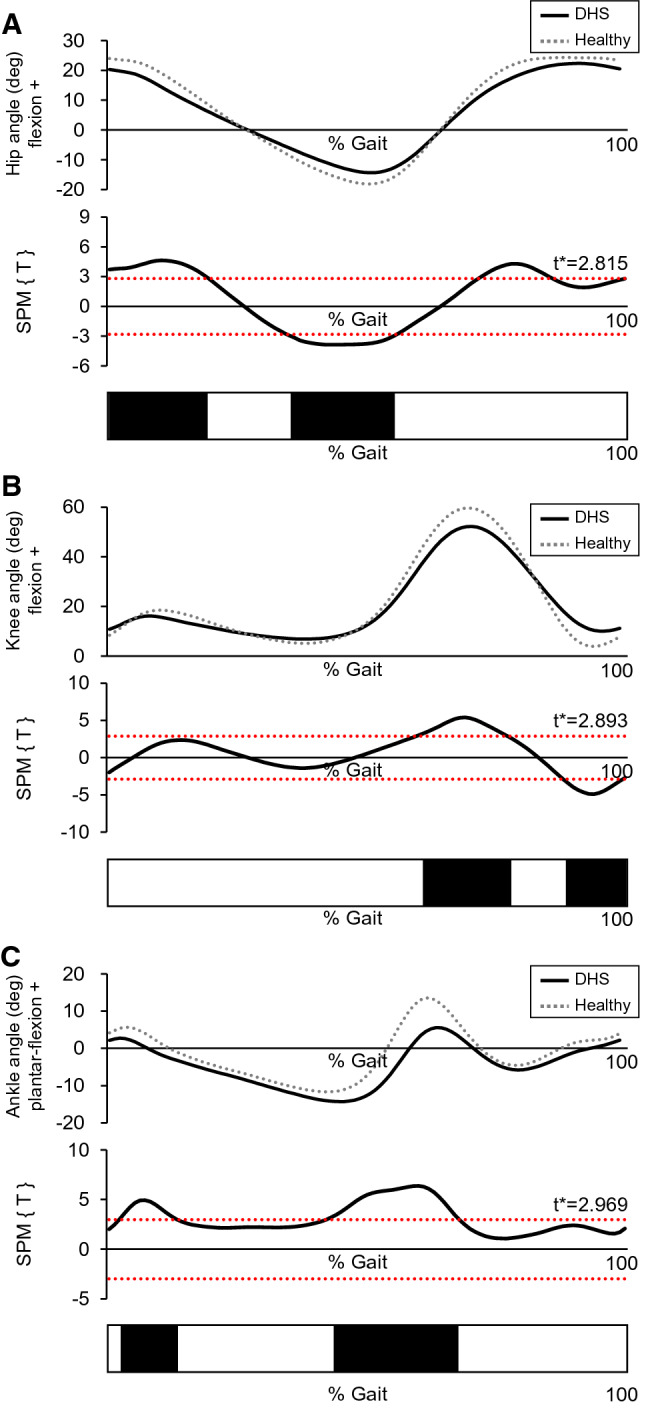
The whole gait cycle waveforms of kinematics in both groups. (**A**) Hip joint angle, (**B**) knee joint angle, and (**C**) ankle joint angle. t* indicate the critical threshold. Lower black bars represent a simplified visualization of the significant areas indicated by the SPM{t} statistic. *DHS* Dropped head syndrome.

**Figure 3:**
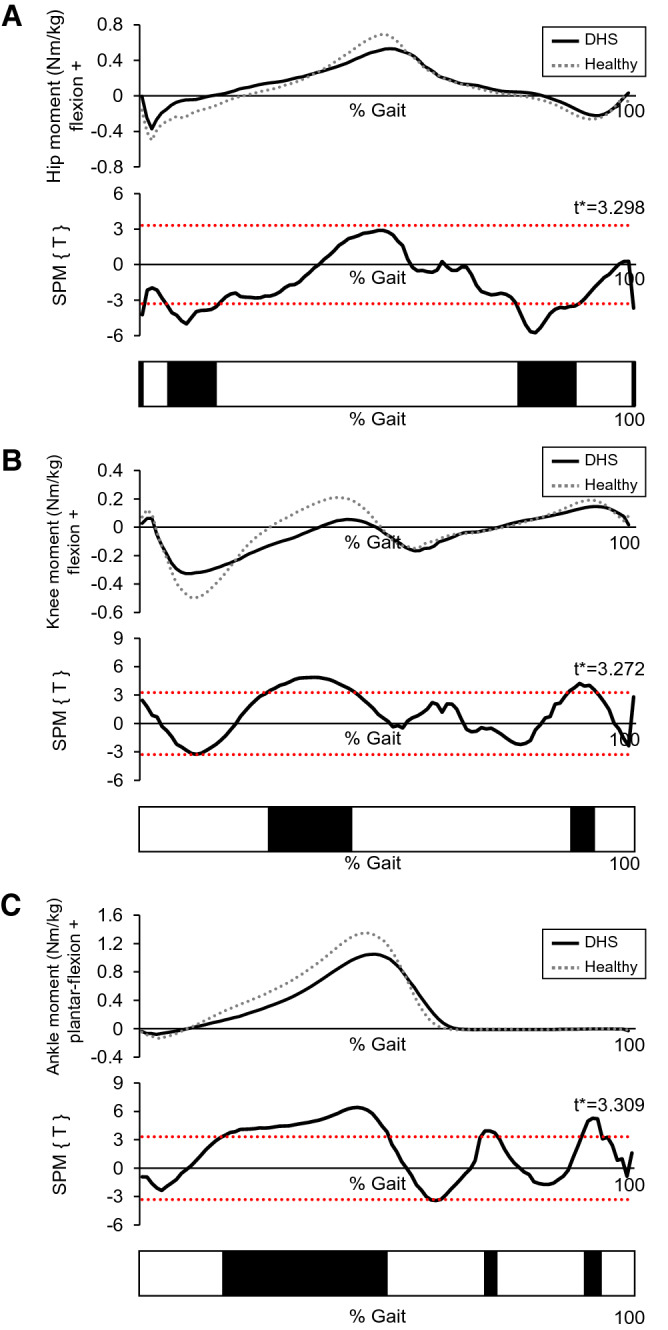
The whole gait cycle waveforms of kinetics in both groups. (**A**) Hip joint angle, (**B**) knee joint angle, and (**C**) ankle joint angle. t* indicate the critical threshold. Lower black bars represent a simplified visualization of the significant areas indicated by the SPM{t} statistic. *DHS* Dropped head syndrome.

The ankle angles were 3–14% and 44–68% during a gait cycle, the ankle moments were 18–51%,71–73% and 91–94%, the knee angles were 61–78% and 90–100%, the knee moments were 27–44% and 88–93%, the hip angles were 1–20% and 36–56%, and the hip moments were 1%, 6–16%, 78–89% and 100%, showing significant differences between the two groups (Figs. 2 and 3). The peak hip-joint extension moment of DHS patients during LR showed no significant difference (*p* = 0.225). The hip-joint moment in patients with DHS changed earlier from extension to flexion than in healthy subjects after initial contact during walking (Table 4, Fig. 3). The peak hip-joint extension moment during SS was smaller than that of healthy subjects (*p* = 0.011). Although the peak hip-joint extension angle of DHS patients during PS was significantly smaller than that of healthy subjects (*p* = 0.024), the peak hip-joint flexion moment showed no significant difference (*p* = 0.370). The knee-joint kinematics and kinetics ware showed no significant difference between the two groups. The ankle-joint dorsi-flexion angles in patients with DHS during SS and PS were significantly larger than that of healthy subjects (*p* = 0.019, *p* = 0.004), and the plantar-flexion moment of ankle-joint was significantly smaller (*p* = 0.001, *p* = 0.028). As a result of comparing the COM-COP distances of the two groups, the AP distances of the DHS group were significantly shorter than those of healthy subjects. (p = 0.007, p = 0.002) (Table 5).

**Table 4:** Comparison of lower limb kinetic and kinematic parameters between the two groups.

	DHS		Healthy		p value
	Mean	(SD)	Mean	(SD)	
**Hip-joint**					
Peak flexion angle during LR (deg)	20.9	(3.7)	24.2	(5.2)	0.084
Peak extension moment during LR (Nm/kg)	0.52	(0.27)	0.68	(0.39)	0.242
Peak extension moment during SS (Nm/kg)	0.07	(0.14)	0.24	(0.18)	**0.018**
Peak extension angle during PS (deg)	15.1	(4.2)	18.7	(5.1)	0.024
Peak flexion moment during PS (Nm/kg)	0.66	(0.16)	0.75	(0.32)	**0.394**
**Knee-joint**					
Peak extension moment during SS (Nm/kg)	0.36	(0.21)	0.52	(0.26)	0.113
Peak extension angle during SS (deg)	6.7	(6.2)	4.4	(5.5)	0.361
Peak flexion angle during PS (deg)	39.9	(6.1)	39.5	(5.6)	0.893
**Ankle-joint**					
Peak dorsi-flexion moment during LR (Nm/kg)	0.12	(0.06)	0.18	(0.09)	0.064
Peak dorsi-flexion angle during SS (deg)	15.3	(2.7)	12.4	(3.1)	**0.022**
Peak plantar-flexion moment during SS (Nm/kg)	1.14	(0.17)	1.39	(0.23)	**0.006**
Peak dorsi-flexion angle during PS (deg)	13.0	(3.1)	8.3	(3.5)	**0.002**
Peak plantar-flexion moment during PS (Nm/kg)	1.05	(0.20)	1.26	(0.22)	**0.027**

Bold figures indicate statistically significant p < 0.05.

*DHS* Dropped head syndrome, *LR* loading response, *SS* single stance, *PS* pre-swing, *SW* swing.

**Table 5:** Comparison of maximum distance between COM and COP.

	DHS		Healthy		p value
	Mean	(SD)	Mean	SD	
ML distance	0.079	(0.019)	0.075	(0.015)	0.573
AP distance (while COP is behind COM)	0.139	(0.040)	0.192	(0.048)	**0.007**
AP distance (while COP is ahead of COM)	0.108	(0.037)	0.169	(0.047)	**0.002**

Bold figure indicate statically significant p < 0.05. Unit is meter.

*COM* Center of mass, *COP* Center of pressure, *DHS* Dropped head syndrome, *ML* Medio lateral, *AP* Anterior–posterior.

## Discussion

A good understanding of gait characteristics provides useful information for appropriate assessment and intervention programs for certain older patients and patients with gait disorders. One of the major features of gait in DHS patients that we first revealed was slow speed and short COM-COP distance. Walking speed is generally evaluated as an outcome indicator of the functional and walking abilities of the elderly^14–16^. These results suggest that DHS patients have lower walking ability than healthy individuals of the same age group. Cervical paraspinal muscle activity is known to be related to walking speed^17^. Abnormal cervical paraspinal muscle activity has been reported in DHS patients^18^ and may be related to the slow walking speed characteristic of DHS patients revealed in this study. A walking speed of 1.0 m / sec or less is also an index of sarcopenia diagnostic criteria^19^, and 92% of DHS patients correspond to this walking speed compared to 25% of healthy subjects who participated in this study. The decrease in walking speed in DHS patients may be caused by weak muscle strength not only in the neck but also in the trunk and lower limbs. A study of patients of similar age to DHS patients who participated in a previous study reported a 70% prevalence of sarcopenia^20^. In addition to loss of neck muscles, loss of total-body skeletal muscle may have affected the gait characteristics of DHS patients, such as slow speed and short stride length. Ferrandez, et al. reported that the double limb support time increased as walking speed decreased^21^. DHS patients also showed similar results to their reports, which suggest that the patient's slower gait is a conservative strategy for increase in stability. Furthermore, the AP distance of COM-COP in DHS patients was significantly shorter than that in healthy subjects (p = 0.007, p = 0.002). It has been reported that people with disabilities who are unbalanced walk with a shorter COM-COP distance^22^. The stride length of DHS patients, which is significantly shorter than that of healthy subjects, also affects the reduction of the amount of AP movement of COP. Keeping the COM close to the COP reduces the moment arm of the limb and reduces the muscle effort required to maintain balance^23,24^. It was revealed that DHS patients walked with more emphasis on balance control than healthy subjects. Quantitative assessment of spatiotemporal gait parameters may help to understand the balance ability of DHS patients.

Alignment of the cervical spine and other spinal columns results in compensatory adaptive changes^3,7^. The results of the radiographic parameters in this study also showed large lumbar lordosis and the proportion of patients with negative C7–S1 SVA (75%), showing a compensatory adaptive posture. The gait analysis data also showed that the thorax backwards tilt angle of DHS patients was significantly larger than that of healthy subjects. We believe that this is a compensatory response to horizontal gaze deficits resulting from the dropped head by tilting the thorax backwards while walking as well as in a static standing position in DHS patients. Flexion of the head, which weighs approximately 7% of the total body weight, can exacerbate the forward shift of the COM, jeopardize standing stability, and make it difficult to recover from unexpected perturbations^6,25^. In DHS patients, tilting the thorax posteriorly during dynamic gait corrects horizontal gaze and improves balance. They have an inadequate ability to correct the relative extension between the thorax and head. The causes include limited range of motion of the cervical spine and muscle weakness. Given the relationship between the dropped head and the posteriorly tilted thorax, reacquisition of these functions may also lead to improved compensatory alignment of standing and walking postures. A prospective study is needed to clarify changes in gait dynamics due to the implementation of exercise programs that improve the dropped head and the use of neck collars that can correct forced neck alignment.

**Figure 4:**
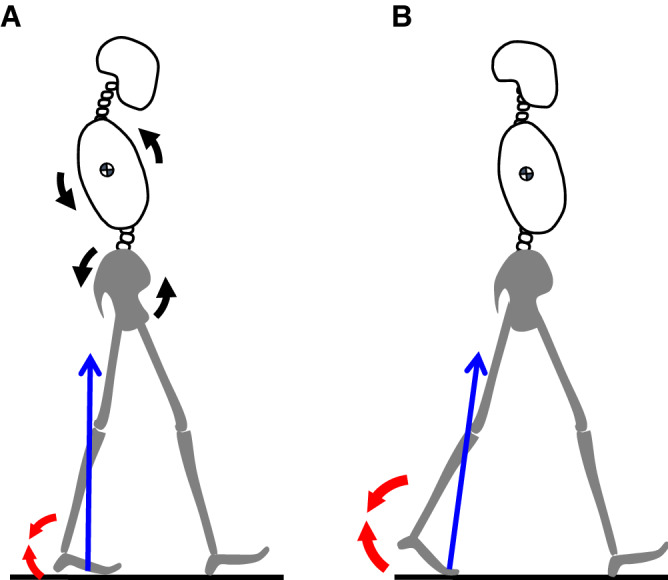
Comparison of the posture and kinetics during gait between the two groups. (**A**) Dropped head syndrome (DHS) group, backward leaning posture of the thorax with an increased ankle-joint dorsi-flexion angle. (**B**) Healthy group, upright posture with a decreased ankle-joint dorsi-flexion angle. Black arrows indicate the backward tilted thorax and pelvis. Blue arrows are the ground reaction force vector. Red arrow is the ankle-joint planter-flexion moment. *DHS* Dropped head syndrome.

In the gait of DHS patients, there were some kinematic and kinetic features of the pelvic and lower limb joints that differed from those of healthy subjects, in addition to the posture in which the upper body mass was displaced posteriorly due to the posterior tilt of the thorax. Firstly, DHS patients had a significantly reduced hip-joint extension angle compared to healthy individuals. Both slow walking speed and reduced hip-joint extension angle reduce hip-joint flexion moment^26^. The walking speed is related to the magnitude of the joint moment, and the cause is the magnitude of the GRF^27^. However, in the results of this study, there was no significant difference in the values of hip-joint flexion moment during stance phase between the two groups. This was the most interesting result as a kinetic feature of the lower limbs. The reduced hip-joint extension angle and slow walking speed were combined with the characteristic walking posture of DHS patients, in which the line of action of the GRF did not pass near the center of the hip-joint during PS due to the anterior pelvic displacement (Fig. 4 and Supplementary Video S1). It is presumed that there was no significant difference in the hip-joint flexion moment of the two groups due to these factors. Secondly, there was a difference between the two groups in 6–16% of the walking cycle in the comparison of the waveform of the entire gait of the hip joint moment, and the peak value of hip-joint extension moment during SS in DHS patients was smaller than that in healthy subjects. These mean that the hip-joint extension moment of DHS patients decreased earlier than that of healthy subjects during LR, and that the flexion moment appeared earlier. It is possible that DHS patients had anterior displacement of the pelvis during walking, because the early reduction of the hip-joint extension moment has been reported as a kinematic feature of walking in the anterior pelvic displacement posture^26^. The basis for this feature may be accounted by the fact that the mean value of C7-S1 SVA was − 11.4 ± 44.8 mm, and that the percentage of patients whose value was negative was 70% in the standing radiographic data. The pelvis is displaced forward as the C7-S1 SVA value decreases^28^. In addition to the effect of gaze compensation, it is also possible that the posterior tilt of the trunk is affected by a decrease in muscle torque of the trunk muscles and hip extensor muscles. It has been reported that the walking posture of a person with reduced hip extensor muscles is characterized by an anteriorly displaced pelvis and a posteriorly tilted trunk^29^. Leteneur et al. found that the extension torque of the hip joint and trunk increased during forward leaning of the trunk, and the flexion torque increased during backward leaning^30^. Their findings suggest that the roles of the trunk and hip muscles are important for postural control of the trunk. Because patients with DHS had abnormal trunk posture and hip torque, a detailed assessment of the trunk and hip extensor muscles may contribute to new insights for clinicians. Thirdly, the kinematics and kinetics parameters of the ankle-joint also showed different characteristics between the two groups. The ankle-joint moment immediately after heel landing is an important factor in shock absorption during walking^31^. The reduced ankle-joint dorsiflexion angle and dorsiflexion moment in DHS patients indicate insufficient shock absorption after heel landing. We speculate that there is a walking characteristic with less impact without the patient noticing it. Finally, throughout one gait cycle, DHS patients had an increased ankle-joint dorsiflexion angle. Especially during LR and the latter half of SS to PS (3–14 and 44–68% gait cycle), the ankle joint angle of DHS patients was significantly larger than that of healthy subjects. Generally, in the case of a healthy person, the heel leaves the floor in the latter half of the SS. The large dorsiflexion angle in DHS patients may have been related to the delayed heel release from the floor. In addition, the ankle-joint plantar flexion moment in DHS patients was smaller than that in healthy subjects. This fact revealed that DHS patients had insufficient ground-kicking using the ankle plantar flexor muscles. The elderly have a lower functional contribution of the ankle during walking than younger people, and the physiological and biomechanical functions of the plantar-flexors decline with age^32^. It is possible that DHS patients have lower ankle function than healthy older people, which may be associated with reduced walking speed. The low ankle-joint plantar flexion moment in DHS patients may be associated with a shortened moment arm due to the short AP distance of COM-COP.

This study had some limitations. Firstly, since this study was conducted with a relatively small number of the patients with DHS, our findings must be further validated in a larger sample size. Secondly, we measured gait under the condition of free speed of the subject. Since walking speed affects joint moments, it can affect the analytical parameters of the lower extremities. In the future, it will be necessary to verify these parameters under a uniform condition using a treadmill or timing light barriers with adjustable walking speed. Finally, the results of this study may not necessarily reflect the most drooping alignment of the head, as there are slight diurnal variations in the degree of head drop in DHS patients. Nevertheless, DHS patients including those with less head drooping conditions are expected to show a tendency to minimize the differences in gait parameters observed between the two groups. A subject of future study is to identify the factors that cause increased head flexion in DHS patients such as postural changes during walking. However, the results of our study cannot conclude that the kinematic and kinetic features of the thoracic and lower limb joints during walking in DHS patients are derived from cervical alignment. Evaluating gait as an outcome in examining DHS patients would provide useful information to explain the improvement in DHS. In the future, it is necessary to investigate the effect of therapeutic intervention on gait parameters of DHS patients.

## Conclusion

We have demonstrated that the walking of DHS patients has kinematic and kinetic characteristics of the lower limb joints and alignment of the thorax and pelvis corresponding to their short stride and slow walking speed.

## Supplementary Information

Supplementary Video S1.

Supplementary Video 1.

## Data Availability

All data generated or analyzed during this study are included in this published article.
