# Supplementary material for: Dynamic alignment changes during level walking in patients with dropped head syndrome: analyses using a three-dimensional motion analysis system
Source: Sci Rep. 2021 Sep 14;11:18254. doi: 10.1038/s41598-021-97329-w (PMC8440518; doi:10.1038/s41598-021-97329-w)
Supplement: Supplementary file 1 — Supplementary Video S1. [file 41598_2021_97329_MOESM1_ESM.pdf]

Supplementary Video S1

Title of the manuscript

Dynamic alignment changes during level walking in patients with dropped head syndrome: analyses using a three-dimensional motion analysis system

Author list

Tatsuya Igawa, Ken Ishii Akifumi Suzuki, Hideto Ui, Ryunosuke Urata, Norihiro Isogai, Yutaka Sasao, Makoto Nishiyama, and Haruki Funao
